# Progress of Gynæcology

**Published:** 1875-05

**Authors:** A. Reeves Jackson

**Affiliations:** Lecturer on Diseases of Women, Rush Medical College, Chicago


ARTICLE II.

### PROGRESS OF GYNÆCOLOGY.

BY A. REEVES JACKSON, M.D.,

LECTURER ON DISEASES OF WOMEN, RUSH MEDICAL COLLEGE, CHICAGO.

1. Myo-Fibroid Tumors of the Uterus, and their Treatment by the Hypodermic Injection of Ergotine. Dr. H. HILDEBRANDT. (*American Journal of Obstetrics*, Feb., 1875.)

2. Death following the Use of Sponge Tents. Dr. DE F. WILLARD. (*Obstetrical Journal of Great Britain and Ireland*, Oct., 1874.)

3. Metro-peritonitis produced by the Use of the ordinary Female Syringe. Dr. THOMAS MORE MADDEN. (*Irish Hospital Gazette*, March 1, 1875; *American Journal Med. Sciences*, April, 1875.)

1. Dr. Hildebrandt has given us another installment of cases, showing the results of the treatment of uterine fibroids by the subcutaneous injection of ergotine; and his extended experience has not lessened his confidence in the efficacy of the remedy.

He first details six cases in which the symptoms of pain, hæmorrhage, etc., were removed, and the tumor diminished in size. Then follow eleven cases, in which but little was accomplished as regards the diminution of the tumor, but in which a material improvement of the symptoms took place; and, finally, two cases in which the treatment exercised no influence whatever, either on the tumor or the symptoms.

Dr. Hildebrandt regards as most favorable for this plan of treatment those cases in which the tumor is so soft and elastic that we have difficulty in distinguishing it by its mere consistence and shape from a dense elastic cyst. Such tumors are common in the uterus, are usually found in young women, and are attended by profuse hæmorrhages and hydrorrhœa. Tumors of this character are generally soft and spongy, and contain more muscular fibres, and are, texturally, similar to the uterus during the early stage of puerperal convalescence. Although he does not agree with the opinion lately expressed by Prof. Spiegelberg to the effect that the complete absorption of an intramural fibroid is possible only when it is not, as usual, enclosed in a capsule, but merges directly without any boundary into the tissue, yet he admits that such are more liable to be rapidly and easily diminished and absorbed than those which are surrounded by a distinct capsule. That is to say, he does not regard the capsule which commonly surrounds a uterine fibroid as an absolute impediment to a cure, because that capsule neither prevents the uniform compression of the tumor, nor is even incapable of absorption itself.

Our author thinks that even with the comparatively small number of observations already made upon this method of treatment, we may accept the following points as conclusively established respecting the prognosis. The treatment is likely to be favorable in its results :

1. When the tumor is richly provided with muscular tissue and possesses the consistence and feel of a tense elastic cyst.

2. When the tumor is submucous.

3. When the walls of the womb are sound, capable of a vigorous contraction, not too much attenuated by dilatation or stiffened by exudation into their substance, and when there is no para- or peri-metritis present.

4. When the tumor is unprovided with a capsule and merges directly without a boundary into the peculiar tissue of the uterus, which anatomical relation of uterine fibroids may be considered most favorable to their complete cure by absorption.

The least promising cases are old anæmic fibroids, composed principally of connective tissue, such as occur in persons of advanced years ; those in which chronic inflammation or its results are present, either in or about the uterus ; those in which the uterine walls are attenuated, or the seat of inflammatory infiltration ; and, those which are subperitoneal. In all the cases belonging to these classes, no appreciable effect was produced by the treatment upon the size of the growth.

The preparation used by Dr. Hildebrandt is an aqueous extract of ergot, prepared according to Dr. Wernich's formula in Berlin. This is mixed with distilled water and glycerine, as follows: ℞. Extract secal. cornut., 3 drams, in aq. destillat., 12 drams, and glycerini puri, 3 drams. Of this he ordinarily injects the contents of a full Pravaz's syringe, containing about 44 drops.*

He makes the injection into the abdominal walls over the site of the tumor, passing the point of the needle deeply into the tissues. In at least one thousand injections of ergotine made by him for various purposes, he has never seen an abscess follow, and only in three clinical cases did this occur. He attributes this immunity from abscess entirely to his mode of procedure, which consists in lifting the skin of the abdomen up in a tolerably high fold by the thumb and finger, and introducing the needle perpendicularly to two-thirds of its length into the apex of the fold, and injecting the fluid very deeply into the subcutaneous cellular tissue—perhaps even into the abdominal muscles. If the injection is made timidly and superficially, a very painful and diffused cutaneous abscess will usually arise.

2. At a meeting of the Obstetrical Society of Philadelphia, held December 3d, Dr. Willard exhibited the uterus of a woman who had died after dilatation of the cervix by sponge-tents. The patient was sterile and earnestly desired offspring, and as the cervix was very narrow a tent was introduced. This slightly dilated the canal, but as it closed up again, a larger one was tried. This produced neuralgic pains, and it was withdrawn and replaced with a smaller one. This was done on a Friday. On the following day the patient, contrary to orders, worked at the sewing machine. On Sunday the

* All the benefits of the hypodermic use of ergot may be obtained by using Squibb's new aqueous extract of ergot, which is soluble in water, free from all irritant matter, and six times the strength of pure ergot. It is carefully prepared for hypodermic use by J. P. Sharp and E. H. Sargent, druggists, of this city.

doctor found her with a tender abdomen, extreme pain, fever, etc. The tent had slipped out of the cervical canal and was found in the vagina. The patient died on the ninth day.

The *post-mortem* examination showed a small amount of serous exudation in the abdominal cavity, and an abscess in the left side of the uterus, containing about an ounce and a half of pus. The parietal layer of the peritoneum was covered with lymph. There had been extensive inflammation of the pelvic viscera; probably at first peri-uterine cellulitis, and then general peritonitis.

During the discussion which followed, four other fatal cases similar to the foregoing were related by Drs. Wilson, Hodge, Smith and Goodell. In all these cases, with a single exception, death took place after a second or third introduction of tents, and Drs. Smith and Goodell expressed the opinion that in this fact was to be found the explanation of the fatal result.

The use of tents for the purpose of dilating the cervix uteri, is unquestionably more dangerous than is usually supposed. While the degree of danger is doubtless increased by the presence of abnormal growths in the womb, and by lack of rest during the period of their use, death has occasionally occurred where the uterus was healthy, and where the patient had been kept perfectly quiet in bed. But tents are too valuable a means of diagnosis to be abandoned on account of the occasional accidents that attend their employment, and hence it is our duty to observe every precaution that may reduce these untoward results to a minimum.

One of these precautions is that alluded to above. We should endeavor to procure all the dilatation necessary by a single tent, or a single batch of tents. The first tent sets up an irritation and congestion of the cervix; its removal produces an abrasion of the mucous surface, and from this is absorbed the decomposing or septic material generated by the subsequent ones. A very excellent plan is that suggested by Dr. Goodell, at the meeting referred to above. He advises the stretching open of the cervical canal by the uterine dilator, and the crowding in of the largest sponge tent possible, and then insinuating around it several small laminaria tents. In this way the required amount of dilatation may frequently be obtained by a single operation.

Tents should never be used so long as inflammation of the pelvic organs, or its results, are present. They should never be used unless in exceptional cases, immediately before or after or during a menstrual period; and, finally, antiseptic vaginal injections, consisting either of solutions of chlorate of potassa, or of carbolic acid, should be used at intervals of three or four hours during the presence of the tent. If these precautions be taken, uterine tents may be used with at least the smallest degree of danger, if not with entire safety.

3. At a recent meeting of the Dublin Obstetrical Society, Dr. Madden related the history of a case showing that the ordinary form of the female syringe is not so harmless an instrument as is commonly supposed. The patient, while using an astringent vaginal injection, five weeks after delivery, had a sudden attack of intense uterine colic, which was followed by severe metro-peritonitis. There was almost complete collapse, accompanied by uncontrollable retching, and for several days the patient's life was in great jeopardy. A portion of the injected fluid had evidently passed through the patulous os into the cavity of the uterus, which was in a state of subinvolution, and thence through the Fallopian tube into the peritoneal cavity.

The foregoing is only one of a number of similar accidents which have been reported within the past few years, and in some of which death has resulted. No instrument is in more common use than the vaginal syringe. Physicians recommend it and patients use it without any idea of its possible danger. Dr. Madden regards it as both objectionable and imperfect; objectionable, because of the possibility of its producing injurious and dangerous effects from the force with which fluid may be thrown into the vagina or even into the uterus; and imperfect, because, to produce any permanent benefit by injections in a case of inflammation or congestion of the cervix uteri, for example, the injected fluid must be kept in contact with the diseased part at a certain uniform temperature for a long time continuously, and this cannot be accomplished with the ordinary syringe, on account of the fatigue attending the working of the instrument, and the irksomeness of the position necessary during its use. To obviate all these inconveniences, Dr. Madden recommends the use of a utero-vaginal irrigator, which operates on the principle of a syphon, and which may be used whenever a vessel of water is obtainable. Such an apparatus is capable of sending a gentle flow of water, plain or medicated, at any desired temperature, into the vagina, for any length of time, and without fatigue to the patient.

The irrigator recommended by Dr. Madden is essentially the same instrument that is so generally known in this country as the Fountain Syringe. Its advantages over the ordinary form of syringe are well known and appreciated among American gynæcologists.

